# Exploring the role of cellular senescence in cancer prognosis across multiple tumor types

**DOI:** 10.3389/fendo.2024.1378356

**Published:** 2024-06-14

**Authors:** Qi Xu, Xiaoying Feng

**Affiliations:** Department of Gastroenterology, Second Affiliated Hospital of Dalian Medical University, Dalian, China

**Keywords:** senescence, cancer prognosis, pan-cancer analysis, senescence-associated secretory phenotype (SASP), nomogram

## Abstract

**Background:**

Cellular senescence is a common biological process with a well-established link to cancer. However, the impact of cellular senescence on tumor progression remains unclear. To investigate this relationship, we utilized transcriptomic data from a senescence gene set to explore the connection between senescence and cancer prognosis.

**Methods:**

We developed the senescence score by the Least Absolute Shrinkage and Selection Operator (LASSO) Cox model. We obtained transcriptomic information of the senescence gene set from The Cancer Genome Atlas (TCGA) program. Additionally, we created a nomogram that integrates these senescence scores with clinical characteristics, providing a more comprehensive tool for prognosis evaluation.

**Results:**

We calculated the senescence score based on the expression level of 42 senescence-related genes. We established the nomogram based on the senescence score and clinical characteristics. The senescence score showed a positive correlation with epithelial-to-mesenchymal transition, cell cycle, and glycolysis, and a negative correlation with autophagy. Furthermore, we carried out Gene Ontology (GO) analysis to explore the signaling pathways and biological process in different senescence score groups.

**Conclusions:**

The senescence score, a novel tool constructed in this study, shows promise in predicting survival outcomes across various cancer types. These findings not only highlight the complex interplay between senescence and cancer but also indicate that cellular senescence might serve as a biomarker for tumor prognosis.

## Introduction

No one can escape from facing senescence as it is a natural process. Hayflick and Moorhead firstly used the concept of “Hayflick limit” to describe cellular senescence, which means proliferating cells have limited proliferative capacity (1). When cells are exposed to stimuli, including noxious (e.g., DNA damage) or non-harmful (e.g., developmental signals) stimuli, they are more likely to enter a state of senescence. Senescent cells release complex cell-specific bioactive molecules, like cytokines, chemokines, growth factors, and proteases. These molecules, known as the senescence-associated secretory phenotype (SASP) (2), profoundly affect neighboring cells, the extracellular matrix, and immune function.

As tumor incidence increases with age, tissue and cell senescence may play a significant role in driving tumor progression. This phenomenon has sparked the interest of researchers exploring the relationships between senescence and cancer (3–10). Senescence and tumor exhibit similar biological characteristics, such as genomic instability, epigenetic changes, and persistent inflammation (11). These common biological characteristics suggest a continuous interaction between senescence and tumor progression. Moreover, this interaction could potentially accelerate the advancement of tumors (12). Senescent fibroblasts, for instance, promote epithelial cell growth and tumor formation (10). Additionally, studies have revealed that hepatocellular carcinoma patients with elevated levels of senescence-related gene expression tend to have shorter survival rates compared to those with lower levels (13). Further research indicates that SASP can either directly or indirectly accelerate tumor progression by promoting tumor cell growth, invasion, metastasis, and tumor angiogenesis (7, 8, 14–16). Besides promoting cancer development, senescence also hampers immune cell function, resulting in an immunosuppressive microenvironment (17).

However, some studies have also shown that senescence can also inhibit tumor progression (18–21). It has been confirmed that CSDE1 induces cell aging by inhibiting YBX1 and inhibits the expression level of tumor-related proliferation markers (22). Schleich et al. found that the high expression level of senescence-related genes in mice can predict favorable outcome of diffuse large B-cell lymphoma (DLBC) patients (19). These findings illustrate the double-edged sword nature of senescent cells in tumor progression. Thus, it is challenging to conclude whether senescence is purely beneficial or harmful to tumor progression. Importantly, the beneficial or harmful effects of senescent cells on tumor progression are not the result of single gene expression, but the result of the synergistic effect of multiple senescence-related genes. Consequently, a novel assessment method that considers the expression of multiple senescence-related genes is needed to better understand the impact of senescence on tumor prognosis.

To investigate this, we analyzed RNA-seq data for 32 different tumor types from The Cancer Genome Atlas (TCGA) database. We downloaded the *SenMayo* senescence gene dataset to obtain senescence-related genes (23). The Least Absolute Shrinkage and Selection Operator (LASSO) Cox model was used to derive a senescence score applicable to the pan-cancer dataset. Based on classification by senescence score, patients in the substantial risk group exhibit shorter survival outcomes than those in the minimal risk group. To validate our findings, we used another senescence-related gene set, *FRIDMAN.SENESCENCE.UP*. This approach simplifies the complex relationship between senescence and cancer outcomes. Finally, by combining the senescence score with the clinical characteristics of patients, including tumor tissue type and patient age, we constructed a nomogram to help clinical physicians predict tumor patient prognosis with distinct kinds of tumors, providing convenience for clinical work.

## Materials and method

### Senescence biomarkers

We sourced senescence-related genes from the *SenMayo* dataset and categorized them into three groups based on Dominik Saul et al.’s classification: transmembrane, secreted, and intracellular (23). We validated the expression of these genes in bone biopsies from aged human cohorts. A total of 125 genes constitute the senescence gene set, with 102 genes included in the training set for signature modeling.

### Patients and datasets

In this study, we obtained pan-cancer transcriptomic data from TCGA (*n* = 8,739). The TCGA data were downloaded from USCS Xena (http://xenabroswer.net/hub). We randomly divided the dataset into a training set and a test set at a ratio of 7:3.

### Construction of the prognostic senescence-related signature

The workflow was as follows: (1) We performed univariate analysis based on the training set. The prognostic genes (*p* < 0.0001) remained for the following analysis (**Supplementary Table S1**). (2) We used the LASSO algorithm for further screening of prognostic genes. (3) We used the Cox proportional hazard regression model with stepwise for the final model fitting. We calculated the senescence score for each patient as follows: senescence score = coefficient *expression of genes (*SenMayo*: 0.026*WNT16 + 0.037*VGF+-0.128*TUBGCP2+-0.068*TNFRSF10C+0.05*SPP1+-0.033*SERPINE2 + 0.135*SERPINE1+-0.107*SCAMP4 + 0.106*PLAUR+-0.041*PLAU+0.221*PIGF+-0.121*NAP1L4+-0.034*MMP9+-0.044*MMP2 + 0.182*MMP14+-0.138*LCP1 + 0.105*ITPKA+-0.063*ITGA2+-0.038*IQGAP2 + 0.033*IL7+-0.045*IL1B+0.03*IL1A+0.034*IGFBP3 + 0.049*IGFBP2+-0.084*ICAM3 + 0.044*ICAM1 + 0.052*HGF+-0.085*ETS2 + 0.067*EREG+0.037*EGFR+-0.035*EDN1 + 0.052*CXCR2 + 0.054*CXCL3+-0.056*CXCL2 + 0.034*CXCL10 + 0.041*CSF1+-0.038*CCL2+-0.037*CCL13 + 0.066*C3 + 0.023*BMP2+-0.038*AREG+-0.027*ANGPTL4. *FRIDMAN.SENESCENCE.UP*, a gene set of senescence-related genes, originated from Fridman [29]: -0.07*CD44 + 0.17*CDC25B+-0.056*CITED2 + 0.22*CLTB+ -0.054*COL1A2 + 0.13*CCNB1 + 0.198*E2F4 + 0.111*EIF2B1 + 0.088*FN1 + 0.095*HPS5 + 0.081*HTATIP2 + 0.03*F3 + 0.041*IGFBP2 + 0.107*IGFBP3 + 0.076*WISP1+-0.055*IGF1R+0.064*MORF4 + 0.059*MYC+-0.051*NDN+0.092*OPTN+-0.044*CDKN2B+-0.139*CDKN2D+0.082*MAPK14+-0.151*NOLC1 + 0.201*PEA15 + 0.055*RAB31 + 0.111*RAB5B+-0.313*RABGGTA+0.256*RAC1+-0.119*S100A11 + 0.024*SERPINB2+-0.085*STAT1 + 0.039*TERT+0.043*TFAP2A+0.033*THBS1 + 0.049*TNFAIP2+-0.065*TNFAIP3. Tumor samples were classified into high-risk and low-risk groups according to the median senescence score.

### *Z*-score evaluation of biological processes

The *z*-score, as introduced by Lee et al. (24), is employed to estimate the activity of specific pathways by amalgamating the expressions of feature genes. We analyzed gene sets associated with glycolysis (sourced from https://www.gsea-msigdb.org/gsea/msigdb/human/geneset/WP_GLYCOLYSIS_IN_SENESCENCE), autophagy (25), and epithelial-to-mesenchymal transition (EMT, sourced from Tian-jian Yu et al.) (26), and we analyzed cell cycle (27) using the GSVA implementation in the R package. The resulting values for each gene set were designated as glycolysis *z*-score, autophagy *z*-score, EMT *z*-score, and cell cycle *z*-score, respectively.

### Differentially expressed genes

We defined differentially expressed genes (DEGs) as those with a log2 fold change (|log2FC|) of 1 or greater and a false discovery rate (FDR) *p*-value of less than 0.05. We used the limma R package for this analysis.

### Statistical analyses

We performed all statistical analysis using R version 4.2.2. We assessed patients’ survival outcomes in the low-risk and high-risk groups using Kaplan–Meier analysis. To assess the clinical utility of each model, we employed decision curve analysis (DCA), a method originally proposed by Vickers and Elkin (28). We evaluated prognostic performance using time-dependent receiver operating characteristic (ROC) curve analysis. We compared the areas under the curves (AUCs) of the senescence score and the nomogram model. For all analyses, we considered a *p*-value of less than 0.05 to be statistically significant.

## Results

### Identification of the senescence-related signature in pan-cancer

**Figure 1:**
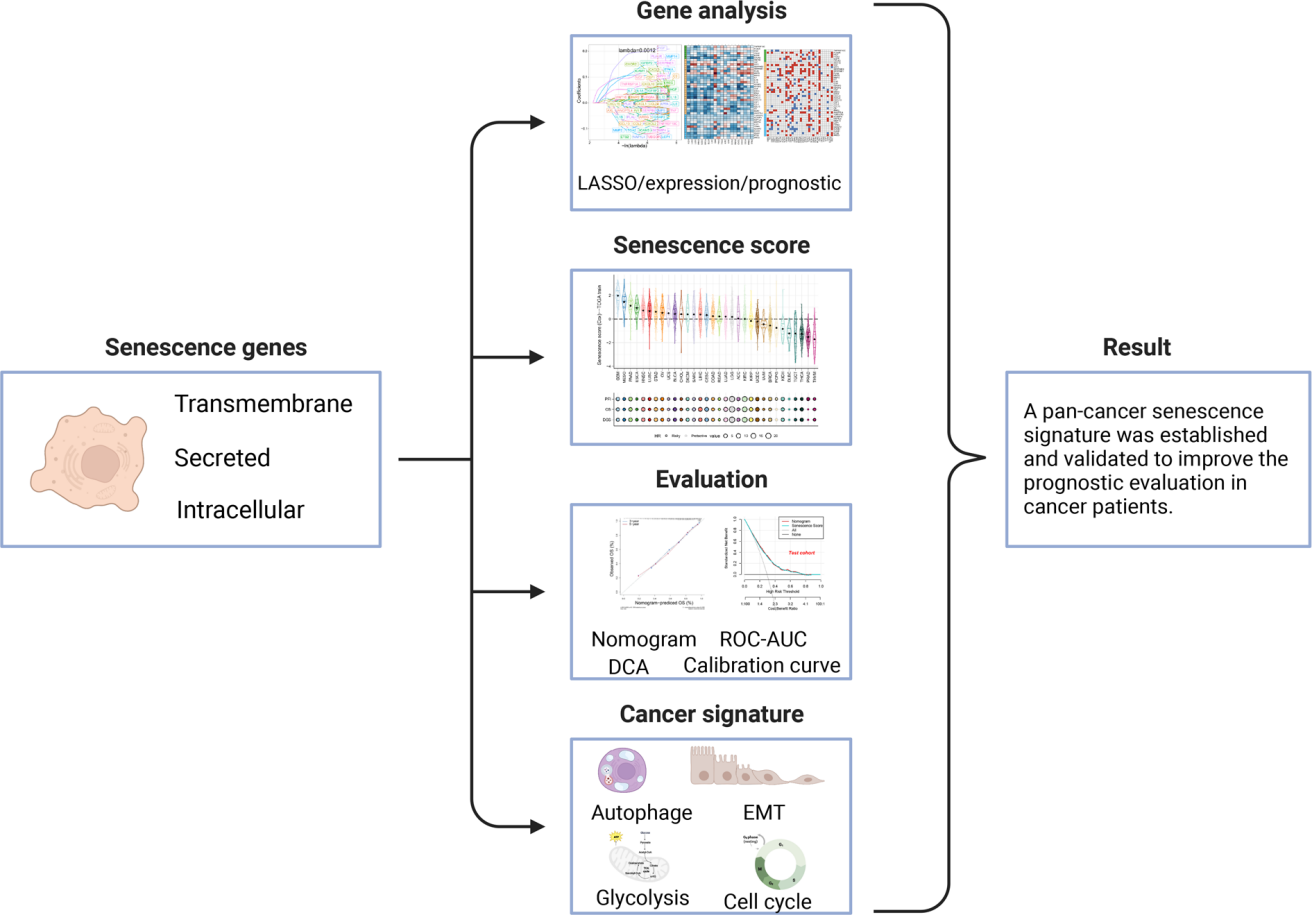
Workflow for analyzing the relationship between senescence-related genes and pan-cancer prognosis.

**Figure 2:**
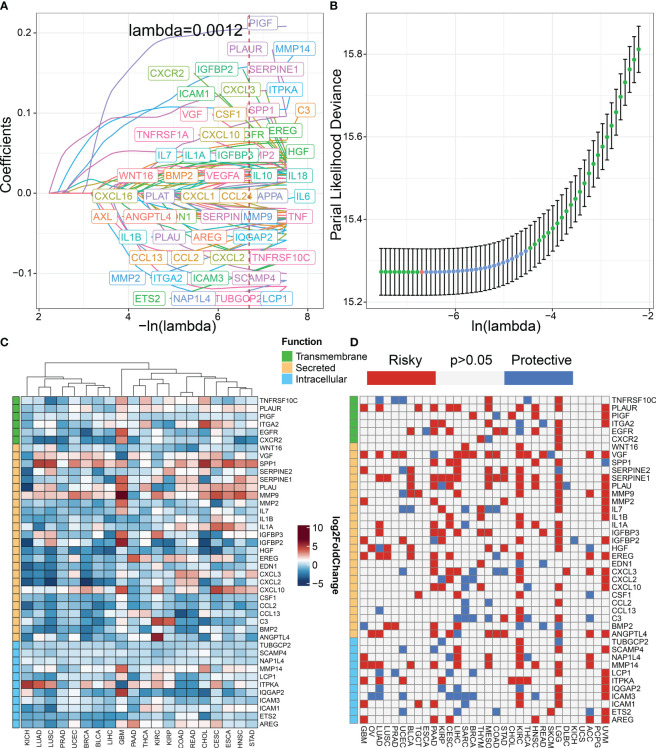
Construction of senescence-related genes in pan-cancer. **(A)** LASSO regressions were employed in the senescence-related signature identity. Coefficient profile plot of predictors was performed against the log (λ) sequence. **(B)** LASSO regression model cross-validation plot. A vertical line was drawn at the optimum with the minimum criterion. Forty-two variables were selected when the most available parameter value λ = 0.0012. **(C)** Compared with normal tissues, the expression level of genes in various tumor types according to TCGA datasets. Red indicates genes with high expression in tumors. Blue indicates genes with low expression in tumors. **(D)** Univariable Cox analysis was employed according to TCGA datasets. Red indicates risky genes in tumor prognosis (HR > 1), while blue indicates protective genes (HR < 1).

The workflow for constructing the senescence-related signature of pan-cancer is shown in **Figure 1**. First, we performed a univariate analysis on 102 senescence-related biomarkers, retaining 57 genes with a *p*-value of less than 0.0001 (**Supplementary Table S1**). Next, we used these 57 biomarkers for LASSO regression analysis in the TCGA pan-cancer training set (**Figures 2A, B**). Finally, we applied the Cox proportional hazard regression model with stepwise, resulting in the selection of 42 senescence-related biomarkers. We derived the senescence score based on the 42 senescence-related biomarkers’ normalized expression levels.

We classified senescence-related genes into transmembrane, secreted, and intracellular categories based on their functional characteristics (23). To further understand the differences in 42 senescence-related biomarkers between tumor and normal tissues, we investigated their expression levels in pan-cancer versus normal tissues. We discovered that secreted biomarkers (*SPP1*, *SERPINE1*, *PLAU*, and *MMP9*) are highly expressed in most tumor tissues, suggesting the role of the SASP in driving tumor progression (**Figure 2C**). To further understand the impact of senescence-related genes on tumor prognosis, we performed univariate Cox analysis (**Figure 2D**). Our findings indicate that most senescence-related genes have a detrimental effect on tumor prognosis.

### Panorama of senescence score in pan-cancer

**Figure 3:**
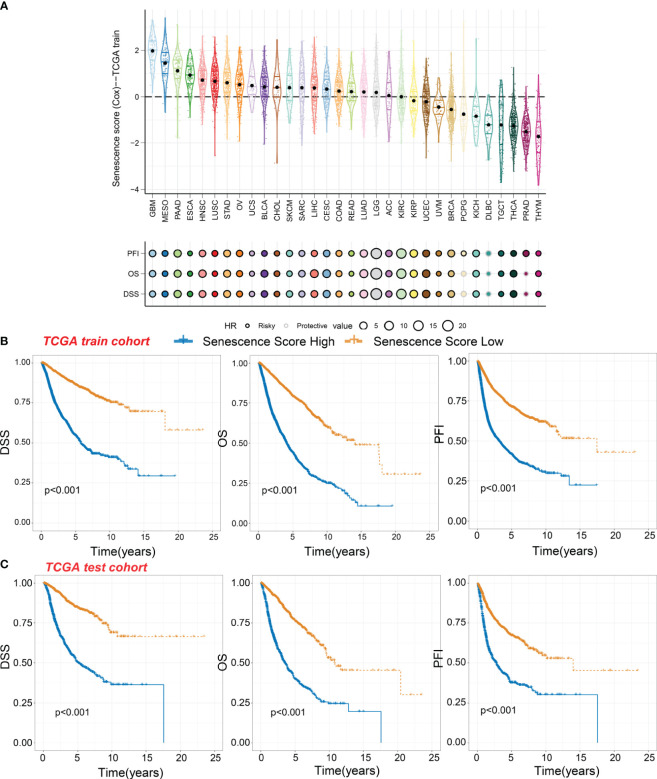
Survival indicators of the senescence score in pan-cancer. **(A)** The senescence scores of various tumor types in the training cohort. Hazard ratio (HR) of progression-free interval (PFI), overall survival (OS), and disease-specific survival (DSS) in various tumor types were calculated. Circles with a black line indicate a risky effect, while a light gray line indicates a protective effect. The value of hazard ratio can be obtained by circle size. **(B)** Patients in the TCGA training cohort were assigned to high or low senescence score groups according to the median senescence score. The DSS, OS, and PFI of different patient groups in the training cohort were shown. **(C)** The DSS, OS, and PFI of high and low senescence score groups in the test cohort were shown.

Significant differences in senescence scores are evident across various organs. Tumors originating from the gastrointestinal tract, such as esophageal cancer, stomach cancer, and colorectal cancer, exhibit higher senescence scores. In contrast, certain gland-associated tumors, including thymoma (THYM), prostate adenocarcinoma (PRAD), and thyroid carcinoma (THCA), display lower senescence scores (**Figure 3A**). The glioblastoma multiforme (GBM), malignant mesothelioma (MESO), and pancreatic adenocarcinoma (PAAD) exhibit the highest senescence scores. Moreover, the senescence score was a risky factor in many cancer types (**Figure 3A**). However, the senescence score was a protective factor in DLBC.

Within the TCGA training cohort, patients were classified into high-score or low-score groups based on senescence scores. Patients with high senescence scores across various cancer types experienced multiple adverse survival outcomes, including short disease-specific survival (DSS), overall survival (OS), and progression-free interval (PFI) (**Figure 3B**). We observed similar results in the test cohort, where the high-score group exhibited worse survival outcomes (**Figure 3C**). This suggests that our senescence score may have a universal applicability in pan-cancer.

**Figure 4:**
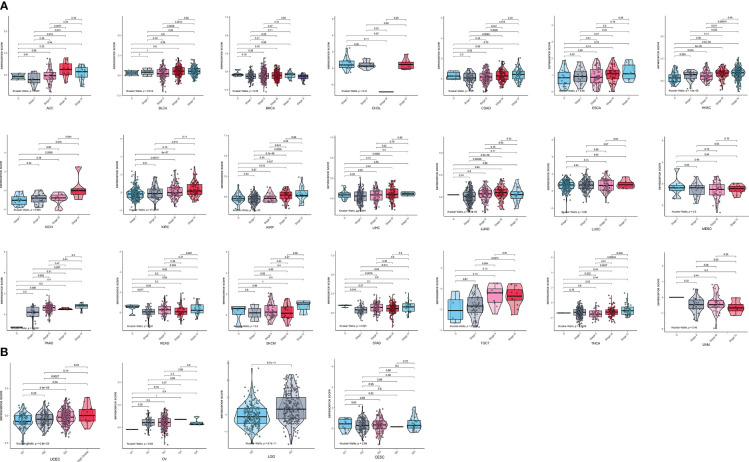
Expression landscape of senescence scores in various stages or grades in pan-cancer. **(A)** The senescence score was analyzed in the main pathological stages in pan-cancer. There is a significant increase in senescence score with increasing tumor stages in adrenocortical carcinoma (ACC), bladder cancer (BLCA), breast cancer (BRCA), colon adenocarcinoma (COAD), head and neck cancer (HNSC), kidney chromophobe (KICH), kidney clear cell carcinoma (KIRC), kidney papillary cell carcinoma (KIRP), liver cancer (LIHC), lung adenocarcinoma (LUAD), pancreatic adenocarcinoma (PAAD), stomach cancer (STAD), testicular germ cell tumor (TGCT), and thyroid carcinoma (THCA). **(B)** The senescence score was analyzed in the main grades in UCEC, OV, LGG, and CESC. There is a significant increase in senescence score with increasing tumor grades in UCEC and LGG.

**Figure 5:**
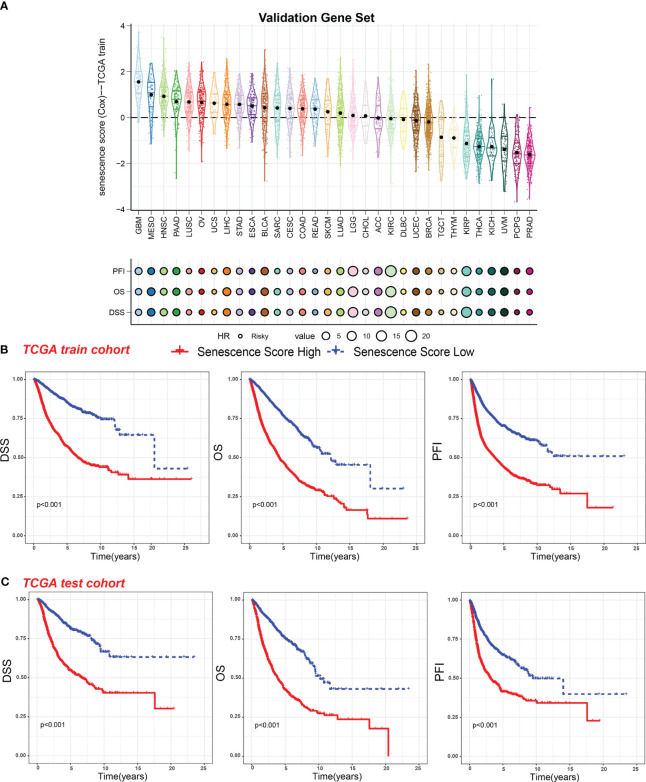
Survival indicators of the validation gene set score in pan-cancer. **(A)** The senescence scores of various tumor types in the training cohort based on the *FRIDMAN.SENESCENCE.UP* gene set. Hazard ratio (HR) of progression-free interval (PFI), overall survival (OS), and disease-specific survival (DSS) in various tumor types were calculated. The value of HR can be estimated by circle size. **(B)** Patients in the TCGA training cohort were assigned to high or low validation gene set score groups according to the median validation gene set score. The DSS, OS, and PFI of different patient groups in the training cohort were shown. **(C)** The DSS, OS, and PFI of high and low validation gene set score groups in the test cohort were shown.

We then analyzed the relationship between senescence scores and different pathological stages in pan-cancer. We examined 21 tumor types and observed a significant increase in senescence scores in 14 of them as tumor stages advanced. They are adrenocortical carcinoma (ACC), bladder cancer (BLCA), breast cancer (BRCA), colon adenocarcinoma (COAD), head and neck cancer (HNSC), kidney chromophobe (KICH), kidney clear cell carcinoma (KIRC), kidney papillary cell carcinoma (KIRP), liver cancer (LIHC), lung adenocarcinoma (LUAD), PAAD, stomach cancer (STAD), testicular germ cell tumor (TGCT), and THCA (**Figure 4A**). With increasing tumor grades, we also observed a significant increase of senescence score in uterine corpus endometrial carcinoma (UCEC) and lower-grade glioma (LGG) (**Figure 4B**). These findings suggest that our senescence score not only is applicable for assessing advanced pan-cancer but also holds significance for identifying early-stage tumors. To validate our findings, we analyzed another senescence signature, termed *FRIDMAN.SENESCENCE.UP* (29). Significant differences in senescence scores from different tumor types were also observed. The GBM and MESO also exhibit the highest senescence scores (**Figure 5A**). Compared with gland-associated tumors, like PRAD and pheochromocytoma and paraganglioma (PCPG), gastrointestinal tract tumors exhibit higher senescence scores. Furthermore, the validation dataset also supports the idea that the senescence score was a risky factor in many cancer types, consistent with senescence scores in the *SenMayo* dataset (**Figure 3A**). In the validation dataset, we classified patients into high- or low-score groups based on their senescence scores. Regardless of whether they were in the training cohort or the test cohort, patients with high scores had worse survival outcomes, including shorter DSS, OS, and PFI (**Figures 5B**, **C**). In that way, this validation indicates that our senescence score could be applicable across various tumor types.

### Establishment and evaluation of a nomogram based on the senescence score for predicting the patient survival

**Figure 6:**
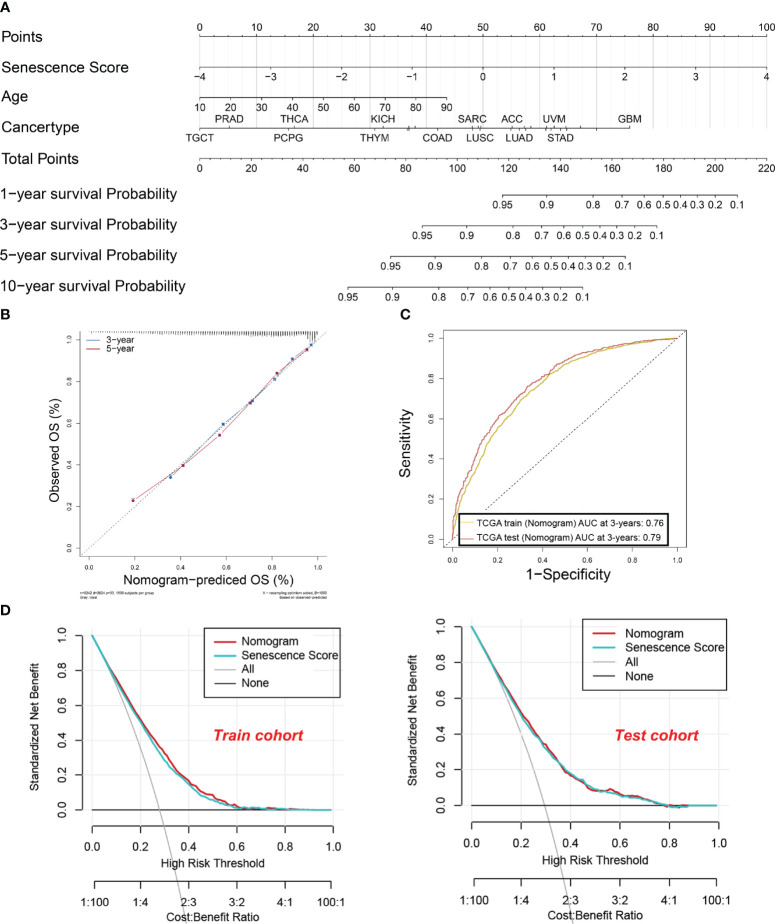
A nomogram were established and evaluated based on the senescence scores for predicting the patient’s survival. **(A)** Nomogram to predict the tumor patient survival. Patients’ clinical characteristics and senescence score were enrolled in the nomogram. Draw a line perpendicular from the corresponding axis of each risk factor until it reaches the line labeled “Total Points”. Sum up the number of points for all risk factors, then draw a line descending from the axis labeled “survival probability” until it intercepts prognosis probabilities. **(B)** Calibration curves for 3-year and 5-year overall survival (OS) in the training cohort. **(C)** The area under the curve (AUC) values for the nomogram in the training and test cohorts at 3 years. The yellow curve indicates. **(D)** Decision curve analysis of the nomogram in the training and test cohorts.

To enhance the practical application of senescence scores in clinical practice, we have developed a comprehensive nomogram (**Figure 6A**). The nomogram integrates senescence scores with clinical characteristics like age and cancer type. In that way, the prediction of patient survival can be easily conducted. The calibration curves for 3-year and 5-year OS (**Figure 6B**) closely align with the standard curve, indicating that the nomogram accurately predicts actual survival probabilities. This alignment indicates that the nomogram accurately predicts the actual survival probabilities. Furthermore, the AUC values for the nomogram are 0.76 and 0.79 in the training and test sets, respectively, underscoring their efficacy in predicting survival rates (**Figure 6C**). Furthermore, DCA in both training and test cohorts shows that the net benefit of combining individual senescence scores with the nomogram is consistently above zero (**Figure 6D**). Although DCA suggests that the nomogram may not offer a significant advantage over senescence scores alone, its utility in clinical practice lies in its ability to help identify high-risk patients with lower survival probabilities, based on tumor type and patient age. For example, as indicated by the nomogram, patients diagnosed with GBM tend to have a lower survival rate compared to those with testicular germ cell tumors (TCGT). This finding underscores the significance of integrating senescence scores with clinical characteristics to enhance the accuracy of prognostic predictions.

### Senescence signature and biological characteristics in pan-cancer

**Figure 7:**
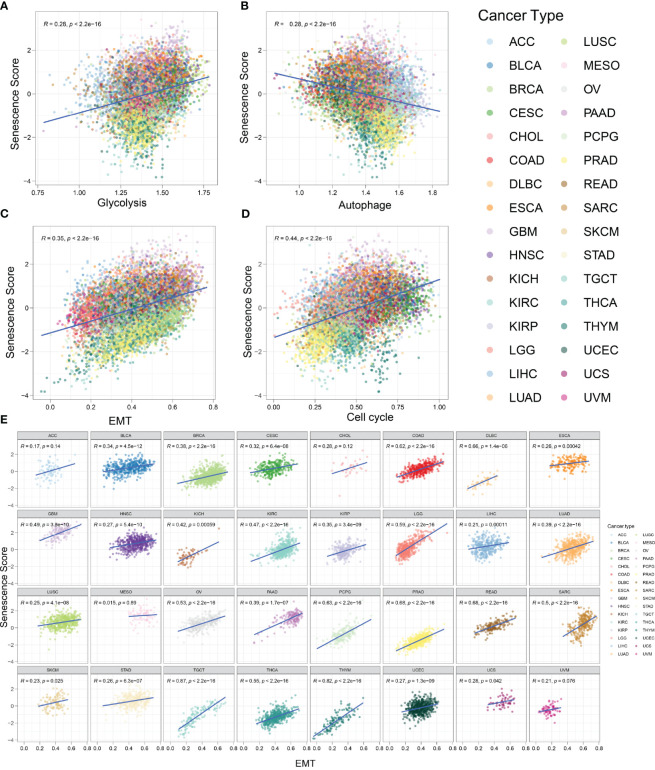
Tumors with high senescence scores represent an aggressive phenotype. **(A)** The activity of glycolysis-related pathways was positively correlated with senescence score in various tumor types. *R* = 0.28, *p* < 0.001. **(B)** The activity of autophagy-related pathways was negatively correlated with senescence score in various tumor types. *R* = 0.28, *p* < 0.001. **(C)** The activity of epithelial-to-mesenchymal transition (EMT)-related pathways was positively correlated with senescence score. *R* = 0.35, *p* < 0.001. **(D)** The activity of cell cycle-related pathways was positively correlated with senescence score. *R* = 0.44, *p* < 0.001. **(E)** The correlation between senescence score and EMT in different tumor types.

Several biological activities occur during the initiation of malignant tumors. As normal cells transform into a malignant state, they require active EMT, rapid and unlimited proliferation, and increased glycolysis frequency (30). It has been reported that senescent cells exhibit high glycolysis rates, which align with the increased demand for proteins and lipids (31). Conversely, autophagy partly suppresses cellular senescence (32). To explore the relationship between tumor characteristics and the senescence signature, we employed the *z*-score algorithm to quantify the activity of cancer-related pathways in various tumor types (**Figure 7**). We found that the senescence score was positively correlated with glycolysis (*R* = 0.28, *p* < 0.001), EMT (*R* = 0.35, *p* < 0.001), and cell cycle (*R* = 0.44, *p* < 0.001). The senescence score was negatively correlated with autophagy (*R* = 0.28, *p* < 0.001). In other words, tumors with a strong senescence state exhibit more active glycolysis, EMT, and cell cycle activities, which represent an aggressive phenotype. Meanwhile, these tumors usually show reduced autophagy, which might promote tumor progression. Furthermore, we evaluated the activity of glycolysis, EMT, cell cycle, and autophagy across a wide range of tumor types. In most tumors, the EMT exhibited strong positive correlations with senescence scores (**Figure 7E**). The correlations between glycolysis and senescence scores across different tumor types are shown in **Supplementary Figure S1**, while those between the cell cycle and senescence scores are depicted in **Supplementary Figure S2**. Most kinds of tumors exhibit positive correlations with senescence scores. Conversely, *z*-scores of autophagy were mostly negatively correlated with senescence scores (**Supplementary Figure S1B**). In summary, our findings indicate that tumors with high senescence scores exhibit more aggressive cellular characteristics.

### Functional analysis in the different senescence score groups

Based on our analysis, we know that tumors with high senescence scores exhibit more aggressive characteristics. It is of great significance whether the biological functions involved in the high senescence score group are related to the mechanism of tumor invasion. To investigate this, we conducted functional analyses to discern differences between high and low senescence score groups. This analysis included biological processes (BP), cellular components (CC), molecular functions (MF), and pathways in the Kyoto Encyclopedia of Genes and Genomes (KEGG).

**Figure 8:**
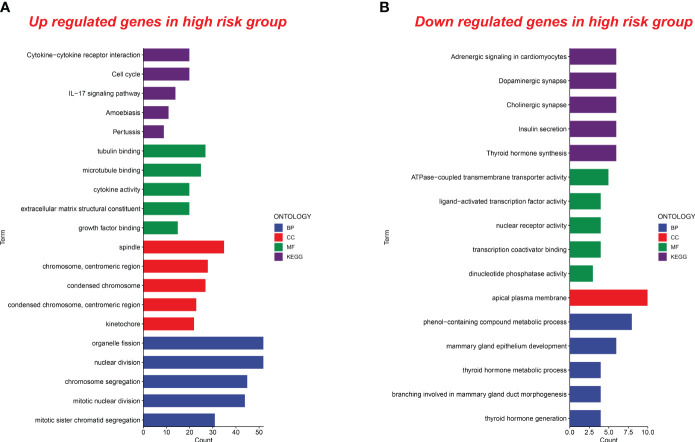
Functional analysis of upregulated and downregulated genes in the high senescence score group. **(A)** Functional analysis of upregulated genes in the high senescence score group was conducted. Biological processes (BP), cellular components (CC), molecular functions (MF), and pathways in the Kyoto Encyclopedia of Genes and Genomes (KEGG) were analyzed. **(B)** Functional analysis of downregulated genes in the high senescence score group was conducted. BP, CC, MF, and KEGG were analyzed.

In the group with high senescence scores, there was a notable enrichment in molecular functions such as tubulin binding, microtubule binding, cytokine activity, extracellular matrix structural constituent, and growth factor binding. Biological processes like organelle fission, nuclear division, chromosome segregation, mitotic nuclear division, and mitotic sister chromatid segregation were also enriched. Upregulated cellular components included the spindle, chromosome centromeric region, condensed chromosome, and kinetochore. Additionally, KEGG signaling pathways showed significant enrichment in cytokine–cytokine receptor interaction, cell cycle, IL-17 signaling, amoebiasis, and pertussis (**Figure 8A**).

In the high-risk group, we observed a downregulation of genes associated with pathways such as adrenergic signaling, dopaminergic synapse, cholinergic synapse, insulin secretion, and thyroid hormone synthesis. Molecular functions like ATPase-coupled transmembrane transporter activity, ligand-activated transcription factor activity, nuclear receptor activity, transcription coactivator binding, and dinucleotide phosphatase activity were also downregulated. Additionally, biological processes and cellular components related to the metabolic processing of phenol-containing compounds, mammary gland epithelium development, thyroid hormone metabolism, mammary gland duct morphogenesis, thyroid hormone generation, and the apical plasma membrane showed decreased activity in the high-risk group (**Figure 8B**). These findings contribute new insights for future research into related biological mechanisms.

## Discussion

Recently, researchers have focused on the relationship between senescence levels and tumor development to understand tumor progression mechanisms. Wu et al. analyzed the results of single-cell sequencing and found that the number of senescent endothelial cells in the tumor microenvironment was significantly increased. They also found that senescent endothelial cells were notably correlated with tumor prognosis (33). The tumor environment contains various cell types, including immune cells, cancer-associated fibroblasts, and other tissue-resident endothelial cells, all of which play roles in tumor progression. For example, senescent fibroblasts promote tumor growth and invasion by secreting cytokines (10). In addition, senescent T cells may also exhibit impaired ability to eliminate tumor cells (34). To account for the comprehensive impact of various cell types in the tumor microenvironment, we established a senescence score using the TCGA bulk-RNA datasets. Pan-cancer patients were divided into substantial risk and insignificant risk groups according to the senescence score. Patients in the considerable risk group exhibit significantly shorter survival periods compared with patients in the insignificant risk group. Furthermore, patients with high senescence scores exhibit an aggressive phenotype: more active glycolysis, EMT, cell cycle, and reduced autophagy.

Zhao et al. obtained sequencing and clinical information datasets for tumor and normal tissues from the TCGA and the Genotype-Tissue Expression (GTEx) (35). With the help of ssGSEA, they also obtained their senescence score. The senescence score they obtained were significantly higher in 13 types of tumors compared to normal tissues. However, we did not compare the differences in senescence scores between tumors and normal tissues. Instead, we observed a significant increase in our senescence score with increasing tumor stages in 14 tumor types (**Figure 4**). In contrast, Zhao’s senescence scores only showed significant correlation with staging in four types of tumors: KIRP, KICH, UCEC, and PAAD. Therefore, our senescence scores have a broader application advantage in predicting tumor progression.

Given the different roles of senescence in various tumor types, predicting tumor prognosis based on senescence levels is challenging (36–38). Senescence scores developed by Zhao et al. were likely to be a risk factor in seven cancer types, while it seems to a protective factor in THYM and HNSC. Meanwhile, Wang et al. developed a senescence score predictor (CS predictor), with the help of machine-learning algorithm (39). The CS predictor exhibits consistency in predicting the prognosis across various tumor types. Patients with a lower CS predictor exhibit significantly worse survival. However, Wang et al. did not provide tumor prognosis predictions for the individual. Our research integrates senescence scores with clinical characteristics to construct nomograms, offering a convenient tool for predicting prognosis in various tumor types, thereby extending the application of senescence scores and providing valuable insights for clinicians.

SASP was originally proposed by Coppe et al. in 2008 (14). They found that senescent cells secrete a set of factors associated with inflammation and tumor progression. These factors were also named SASP (14). Senescent cells in the tumor microenvironment also secrete SASP, involving tumor growth and invasion. These factors, termed SASP, engage in multiple physiological processes, including cell proliferation, immune response, programmed cell death, cell cycle regulation, cell adhesion, and immune response (39). Most of these genes are included in our senescence score, such as *WNT16*, *EGFR*, *SERPINE1*, *SERPINE2*, *ICAM1*, *ICAM3*, *MMP*, *IL1A*, *IL1B*, *IL7*, *CCL2*, and *CCL13*. *WNT16* and *EGFR* are critical members of cell proliferation (32). *SERPINE1*, *SERPINE2*, *ICAM1*, and *ICAM3*, among others, are involved in the remodeling of extracellular matrix and the promotion of intercellular adhesion (33). *MMP2*, *MMP9*, and *MMP14* are involved in the degradation of extracellular matrix (34). *IL1A*, *IL1B*, *IL7*, *CCL2*, and *CCL13* are involved in the regulation of tumor immune microenvironment. Our senescence score shows that senescence plays an array of functions in the development of tumors, which is in line with previous investigations (4).

In addition, tumors with senescence scores exhibit more aggressive cell characteristics. Owing to the decreased mitochondrial function, senescent cells exhibit aberrant active glycolysis. Active glycolysis contributes significantly to the Warburg effect observed in tumors, increasing tumor cells’ hypoxic environment adaptability. Furthermore, increased lactate levels in the tumor microenvironment suppresses immune cell functions and helps immune escape. Consequently, these processes promote tumor growth and development (30, 40). EMT leads to the loss of epithelial cell characteristics, like decreased cell adhesion, and the acquisition of mesenchymal cell characteristics. EMT helps tumor cell migration and invasion, one of the main mechanisms of tumor progression (41). Senescent human fibroblasts secrete growth factors and cytokines to promote the transition of premalignant and malignant epithelial cells to mesenchymal cells (10). It has been found that high expression of α-smooth muscle actin in myofibroblasts promote EMT and tumor progression *in vitro* (42). Additionally, autophagy is a fundamental cellular process. It eliminates molecules and subcellular elements, including nucleic acids, proteins, lipids, and organelles, through lysosomal-mediated degradation. Under physiological conditions, autophagy helps maintain cellular and organismal homeostasis. However, in disease states, autophagy is mostly dysfunctional. Decreased autophagy leads to neuromuscular dysfunction (43), while excessive autophagy causes metabolic disorders in senescent adipocytes (44). Thus, we analyzed the correlation between senescence scores and autophagy. We found a negative correlation between senescence scores and autophagy, which is consistent with previous findings (45, 46). Interestingly, in some tumors, such as ACC and BLCA, there is no correlation between autophagy and senescence scores. This may be related to the tissue specificity of autophagy (47). For instance, intestine-specific expression of *atg-18* can rescue the lifespan of *atg-18* mutants, whereas muscle-specific expression of *atg-18* fails to rescue the lifespan of *atg-18* mutants (48). This may be the result of the combined action of autophagy-related gene expression between tumor tissue cells and stromal cells in the microenvironment. It is interesting that senescent cells often exhibit cell cycle arrest, providing a protective mechanism for DNA damage and metabolic stress, while tumor cells often exhibit infinite proliferation, which seems to show a contradiction between senescence and tumors. However, senescent cells and tumor cells both show a neglect of cell cycle checkpoints, providing a solution for resolving the contradiction (49).

Nevertheless, there are a few constraints to our study. To begin, because of the difficulty in gathering sufficient clinical samples, we were unable to verify the senescence score with external clinical data. Secondly, owing to unfinished patient treatment data in the database maintained by the TCGA and the challenge in gathering patient treatment data, our study overlooked the impact of treatment on the prognosis of tumor patients, which limits its applicability. In our study, we analyzed bulk RNA sequencing datasets, which made it challenging to determine whether the expression of senescence-related genes originated from tumor cells or other senescent cells within the tumor microenvironment. In that way, it limits further exploration of senescence-related genes. Therefore, we can collect clinical samples in the future, classify senescent cells in the tumor microenvironment, and systematically analyze which senescent cells have a critical role in tumor prognosis. This will provide a strategy for targeting treatment of tumor patients.

## Conclusion

In summary, our findings shed new light on the role of senescence-related genes in tumor development mechanisms. The senescence score established during this study is important for predicting the prognosis of pan-cancer patients. Future research should aim to validate these findings and investigate prospective therapeutic approaches targeting senescence-related pathways.

## Data availability statement

Publicly available datasets were analyzed in this study. This data can be found here: https://portal.gdc.cancer.gov/.

## Ethics statement

Ethical approval was not required for the study involving humans in accordance with the local legislation and institutional requirements. Written informed consent to participate in this study was not required from the participants or the participants’ legal guardians/next of kin in accordance with the national legislation and the institutional requirements.

## Author contributions

QX: Writing – original draft. XF: Writing – review & editing.

## Supplementary material

Supplementary Figure 1
**(A)** The correlation between senescence score and glycolysis in different tumor types. **(B)** The correlation between senescence score and autophagy in different tumor types.

Supplementary Figure 2 The correlation between senescence score and cell cycle in different tumor types.
