# Supplementary figures and images for: Exploring the role of cellular senescence in cancer prognosis across multiple tumor types
Source: Front Endocrinol (Lausanne). 2024 Jun 14;15:1378356. doi: 10.3389/fendo.2024.1378356 (PMC11211249; doi:10.3389/fendo.2024.1378356)

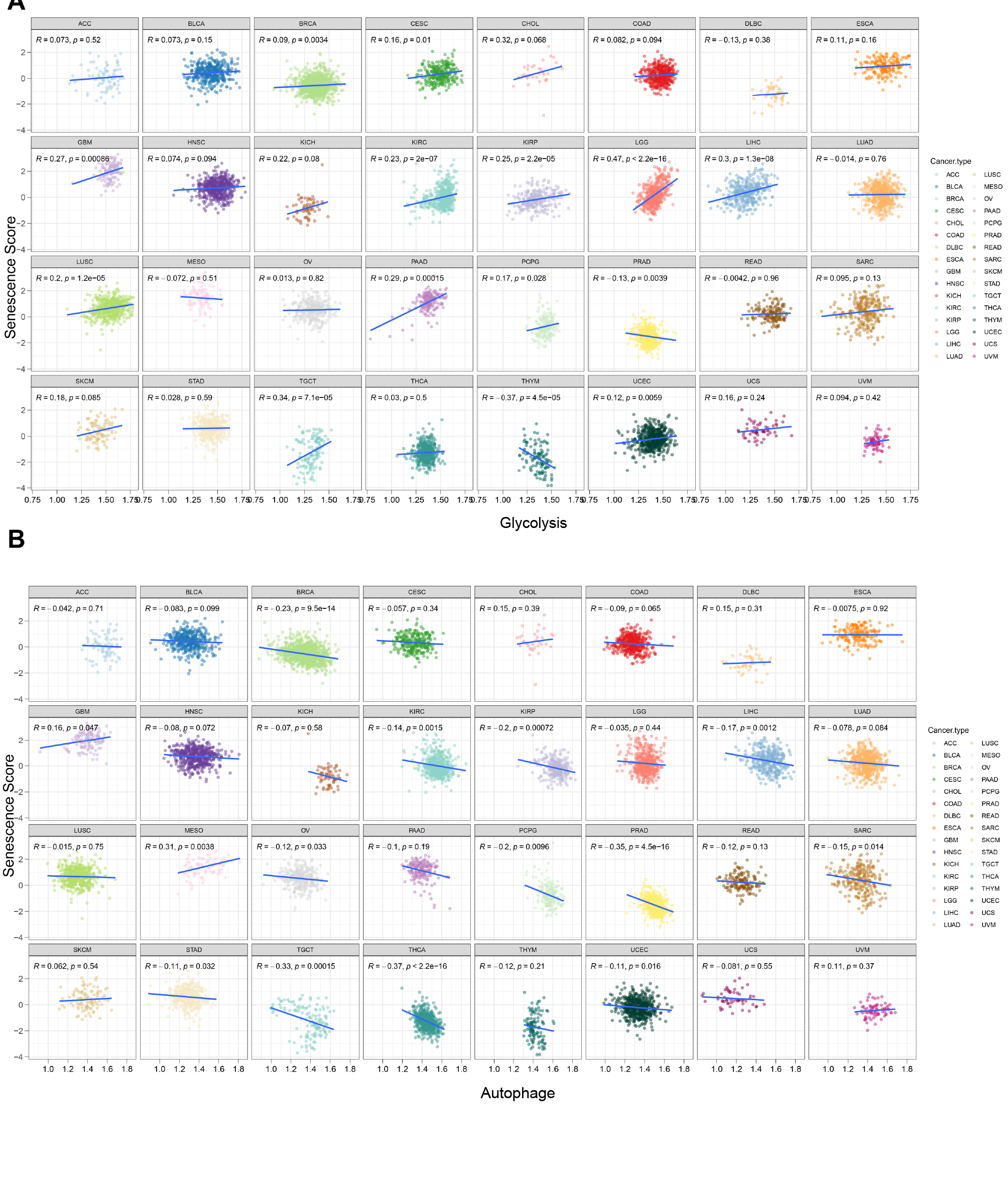

Supplement: Supplementary Figure 1 — (A) The correlation between senescence score and glycolysis in different tumor types. (B) The correlation between senescence score and autophagy in different tumor types. [file Image_1.tif]

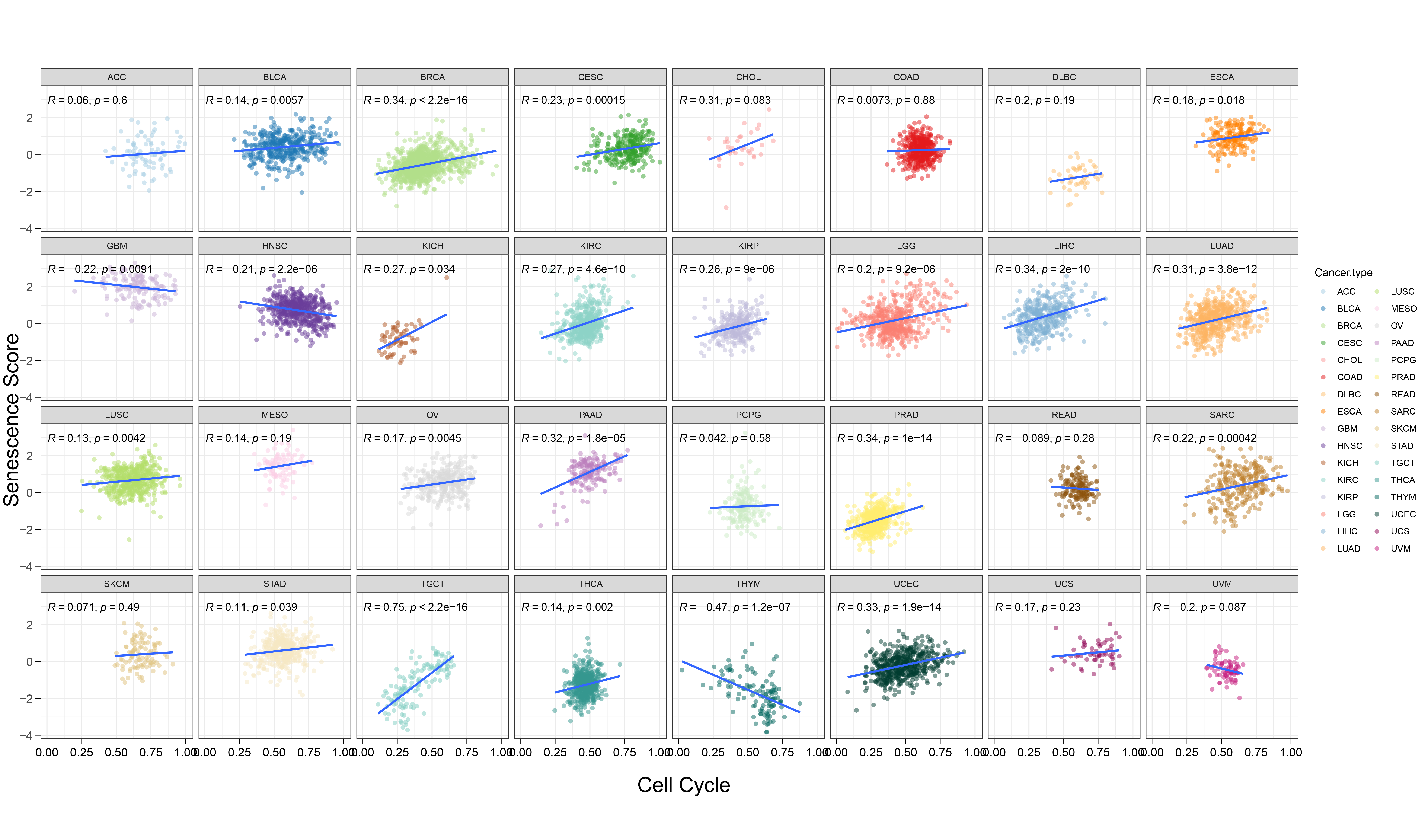

Supplement: Supplementary Figure 2 — The correlation between senescence score and cell cycle in different tumor types. [file Image_2.tif]
